# Dip-Printed Microneedle Motors for Oral Macromolecule Delivery

**DOI:** 10.34133/2022/9797482

**Published:** 2022-07-20

**Authors:** Xiaoxuan Zhang, Guopu Chen, Lijun Cai, Lu Fan, Yuanjin Zhao

**Affiliations:** ^1^Department of Rheumatology and Immunology, Nanjing Drum Tower Hospital, School of Biological Science and Medical Engineering, Southeast University, Nanjing 210096, China; ^2^Oujiang Laboratory (Zhejiang Lab for Regenerative Medicine, Vision and Brain Health), Wenzhou Institute, University of Chinese Academy of Sciences, Wenzhou 325001, China; ^3^Institute for Stem Cell and Regeneration, Chinese Academy of Science, Beijing 100101, China

## Abstract

Micromotors have demonstrated values in drug delivery, and recent attempts focus on developing effective approaches to generate functional micromotors to improve this area. Here, with the integration of microfluidic droplet printing and wettability-induced drawing photolithography, we present an innovative spatiotemporal serial multistep dip-printing strategy to generate novel independent microneedle motors (IMNMs) for orally delivering macromolecular drugs. As the strategy combines the advantages of the hydrophilic wettability, extension effects, and capillary effects, the IMNMs with an oblate basement and a needle-shaped head or a core-shell structured multicomponent head can be created by simply printing pregel droplets layer by layer, following with simultaneous wiredrawing and solidification. Owing to the polarized magnetic particles in the bottom basement and the rapidly dissolvable polymers as the middle basement, the resultant IMNMs can respond to magnetic fields, move to desired places under a magnet, penetrate tissue-like substrates, induce head-basement separation, and leave only the needles for cargo release. Based on these features, we have demonstrated that these IMNMs can deliver insulin via intestinal tracts to realize effective blood glucose control of diabetic rabbit models. These results indicate the practical values and bright future of the dip-printing stratagem and these IMNMs in clinical applications.

## 1. Introduction

Micromotors are miniature movable devices which can be self-driven or actuated by external factors such as light, ultrasound, and magnetism [1–3] Benefitting from their small size, the ability to be remotely manipulated, and easy integration with other technologies, micromotors have shown bright prospects in biomedicine, environmental engineering, industrial production, and many other fields [4–8]. To achieve these micromotors, various strategies have been developed, including top-down approaches [9–13] like etching, eroding, and surface coating as well as bottom-up methods [14–17] like chemical synthesis and self-assembling. By these means, a wide range of biomedical micromotors have been created and applied for minimally invasive surgery, diagnosis, cell manipulation, drug delivery, and so on [18–22]. Although with many successes, their current fabrication normally requires complicated chemical reactions or high-precision micro-/nanolithography or molding techniques, bringing difficulties in generalization and commercialization. Besides, most micromotors only have simple structures such as spheroids and quasispheroids, which restricts the extension of their functions and makes them unsatisfactory in some situations of drug administration, etc. Therefore, the development of facile, promotable, and easy-to-operate approaches for producing functional and featured micromotors is highly anticipated.

**Figure 1:**
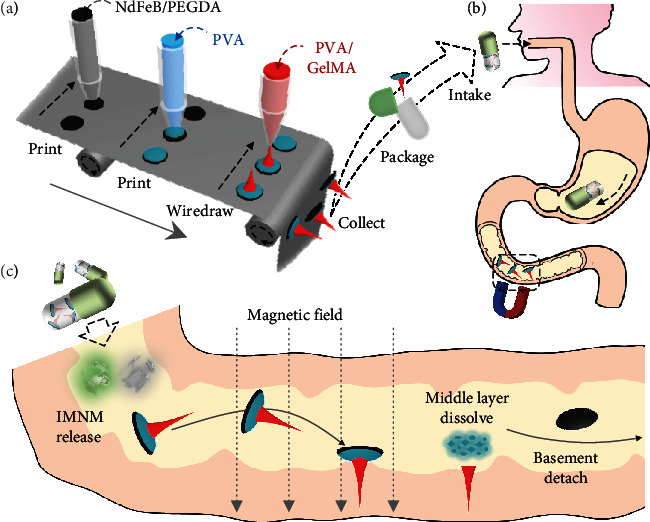
Schematic illustrations showing the fabrication and application of IMNMs. (a) Scheme of the entire fabrication process, which includes printing the basement, printing the middle layer, wiredrawing the needle layer, and packaging the IMNMs in capsules. (b) Scheme of the uptake of IMNM-loaded capsules. (c) Scheme of the capsule breakage, IMNM release, magnetically guided movement and penetration, and needle-basement detachment.

In this paper, we developed an effective dip-printing strategy to produce novel microneedle motors with desired features for intestinal macromolecule delivery, as schemed in Figure 1. As a class of popular and effective technology, printing, especially three-dimensional (3D) printing, has been widely employed in the fabrication of microsized products including microfibers [23], microhelixes [24], microparticles [25–27], diversely shaped microrobots [28], and so on [29]. Generally, the 3D printing is a process of building 3D objects by depositing required materials layer by layer, which mainly includes fuse deposition modelling, direct ink writing, stereolithography, and polyjet strategy [30–32]. Benefitting from the accuracy and controllability of the 3D printing technology, the produced objects show uniform morphology and are highly tunable [33–35]; whereas existing 3D printing methods for micromotor fabrication usually rely on complex and sophisticated optical and micromachining equipment, which is relatively difficult to implement. In addition, the homogeneous structure and material composition of the fabricated micromotors cause limitations in their practical functions and applications such as compound drug delivery and smart controlled release. Thus, generating heterogeneous micromotors with improved printing approaches is still waiting to be explored.

Herein, by integrating traditional microfluidic droplet printing with wettability-induced drawing photolithography, we present a spatiotemporal serial multistep dip-printing strategy for the desired micromotor fabrication. During this process, with the synergies of the hydrophilic wettability, extension effects, and capillary effects, composite objects with an oblate basement and a needle-shaped head could be facilely generated by printing pregel droplets on slides while simultaneously wiredrawing and solidifying. Notably, with repeated dipping and drawing, the head of these composite objects could be multifarious, ranging from single-core to multilayer core-shell structures and from single- to multi-property material compositions. As the magnetic particles were mingled in the bottom basement layer, the composite objects could be endowed with magnetic polarity, making them be flexibly manipulated under magnetic fields and serve as independent microneedle motors (IMNMs). Besides, these IMNMs could be imparted with separable abilities by simply employing rapidly dissolvable polymers as the middle basement layer during the fabrication. Given these features, the IMNMs under a magnet were found to be guided to desired positions, penetrate into tissue-like substrates, and leave only the needles to release cargos, which made them promising in intestinal drug delivery. For demonstration, such IMNMs were loaded with insulin to treat diabetic rabbit models, whose ideal performances indicated their advantages as micromotors in oral drug administration and other biomedical applications.

## 2. Results

**Figure 2:**
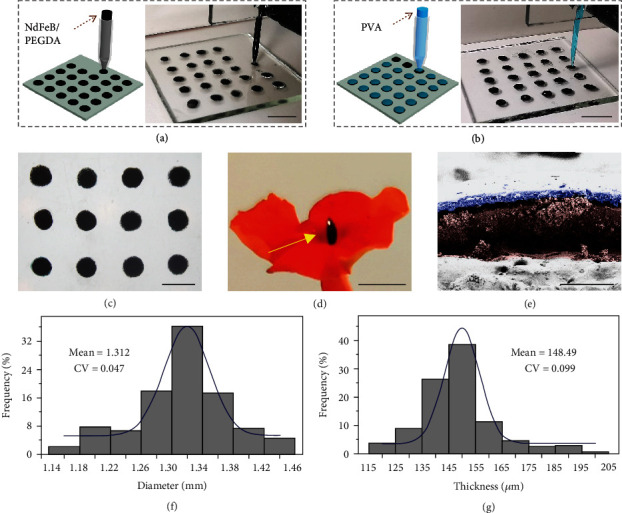
Fabrication and characterization of the double-layer composite. (a) Schematic and digital image of printing the magnetic basement on a glass plate. (b) Schematic and digital image of printing the middle layer on the magnetic basement. (c) Optical image of an array of double-layer composite. (d) Digital image of a double-layer composite standing on a petal under the guidance of a magnet. (e) Pseudocolor SEM image showing the different layers of the double-layer composite. Purple: the middle layer; brown: the basement layer. (f) Diameter distribution of the double-layer composite. (g) Thickness distribution of the double-layer composite. The scale bars are 5 mm in (a, b), 2 mm in (c, d), and 200 *μ*m in (e).

In a typical experiment, the magnetically controlled basement was generated using a capillary microfluidic device. A mixture solution of polyethylene glycol diacrylate (PEGDA) and neodymium-iron-boron (NdFeB) microparticles (~5 *μ*m) was pumped into the tapered round capillary and collected on a shifting glass plate (Figure 2(a)). During this process, the glass plate continuously changed its position, and an oblate droplet array was obtained. These oblate droplets were then solidified by ultraviolet (UV) light to form the magnetic basements. It was found that the sizes of the basements could be flexibly adjusted by changing the flow rate of the mixture solution, as recorded in Figure S1. With the similar approach, polyvinyl alcohol (PVA) solutions were added through another tapered round capillary to cover the surfaces of the basements and were dried to constitute the detachable middle layers (Figure 2(b)). Optical images displayed the circular morphology of the resultant double-layer composites, which were small in size and light in weight (Figures 2(c) and 2(d)). Besides, the basement layer and the middle layer were further revealed clearly by the scanning electron microscope (SEM) image, as shown in Figure 2(e). The energy-dispersive spectrometer (EDS) elemental mapping showed the encapsulation of NdFeB particles inside the basement layers (Figure S2). It should be mentioned that such double-layer composites had satisfactory uniformity, with both the diameter and the thickness centering in a small range (Figures 2(f) and 2(g)).

Resorting to a microfluidic drawing method, the needle layer was assembled with the afore-made double-layer composite. In particular, to construct the needle-like shapes, the materials of the needle layer were required to be viscous enough for wiredrawing. Besides, as the tissue-penetrated needle tips, these needle layer materials should meet the mechanical demands. Given these considerations, we mixed biocompatible methacryloyl gelatin (GelMA) that possessed satisfactory mechanical strength at high concentrations with PVA to increase the viscosity. It was known that GelMA solutions would form covalently cross-linked networks under UV irradiation and that PVA solutions would be gelled through repeated freeze thawing processes with the help of hydrogen bonds; thus, the needle layer prepolymer solution could become double network (DN) hydrogels after experiencing UV solidification and freeze thawing, as shown in Figure S3. For finding out an appropriate formula, the viscosity of the prepolymer solutions and the mechanical strength of the solidified DN hydrogels under different GelMA or PVA concentrations were further investigated. Results showed that the increase of either the GelMA concentration or the PVA concentration would lead to the enhancement of the viscosity, while the PVA concentration had a much more significant impact (Figure S4). Similarly, the increase in both concentrations would result in the improvement in the mechanical strength (Figure S5). Based on these investigations, a mixture of 30% GelMA with 15% PVA was determined to be an optimized condition for the following experiments.

**Figure 3:**
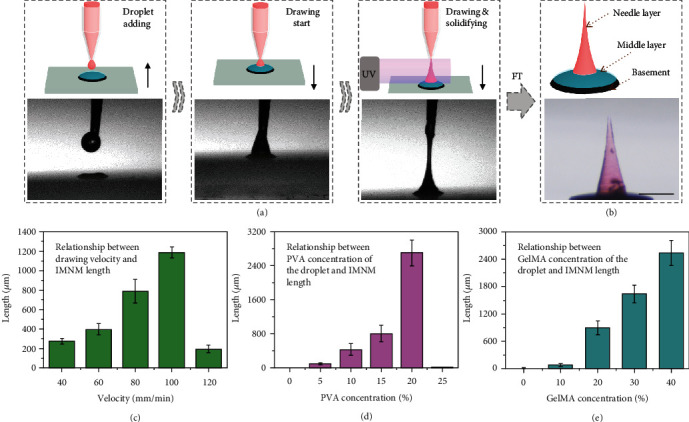
Fabrication and characterization of the IMNMs. (a) Schematics and optical images of the drawing process. (b) Schematic and optical image of the components of the IMNM. The needle layer is dyed red. (c) Influence of drawing velocities on IMNM lengths. (d) Influence of PVA concentrations on IMNM lengths. (e) Influence of GelMA concentrations on IMNM lengths. The scale bar is 500 *μ*m.

During the drawing process, a droplet of the needle layer prepolymer solution was first extruded through a tapered round capillary, and the glass plate carrying the double-layer composite moved up to approach the droplet, as shown in Figure 3(a). After the droplet and the double-layer composite contacted, the glass plate began moving down and the drawing started. When a conical droplet appeared, the UV light was turned on to synchronously carry out drawing and solidifying. This procedure continued until the hydrogel filament broke up. Eventually, the desired IMNM composed of a magnetic basement layer, a detachable middle layer, and a needle layer was obtained by repeated freeze thawing (Figure 3(b)). The upper diameter of the needle tip was measured to be about 30 *μ*m, and the maximum stress that an IMNM could tolerate was more than 0.34 N (Figure S6), which well met the demands for tissue penetration [36]. To realize a better control over the morphology of IMNMs, influencing factors including the drawing velocity, as well as the PVA concentrations and GelMA concentrations of the prepolymer solution were evaluated. It was found that as the drawing sped up, the length of the needle layer first experienced a dramatic increase and then a sudden decrease (Figure 3(c)). The reason could be that the faster drawing velocity allowed the hydrogel filament to stretch longer before complete solidification, while the overquick speed (over 120 mm/min) caused the filament to break earlier than curing. Additionally, the increase of both PVA concentrations and GelMA concentrations contributed to the viscosity of the prepolymer droplet, helping the filament be drawn longer and bringing about a taller needle layer (Figures 3(d) and 3(e)), whereas, when the PVA concentration reached 25 wt%, the prepolymer solution turned gelatinous and could not be lifted up.

**Figure 4:**
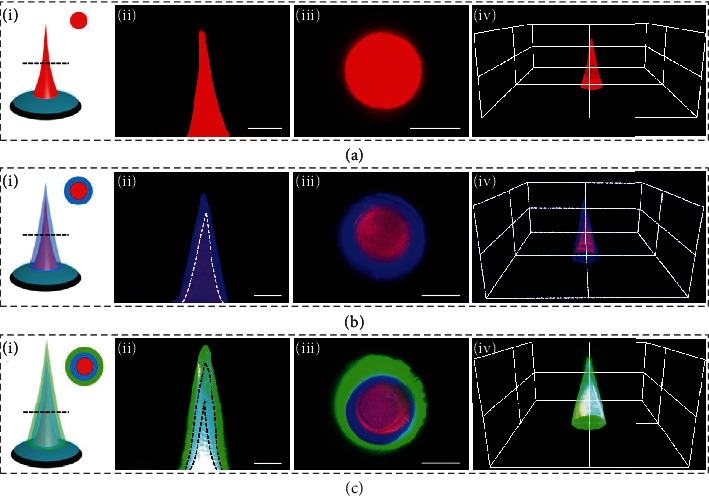
Characterization of core-shell IMNMs. (a) Schematic (i), longitudinal section fluorescence image (ii), cross-section fluorescence image (iii), and 3D reconstruction fluorescence image (iv) of the shell-less IMNM. (b) Corresponding schematic and fluorescence images of the single-shell IMNM. (c) Corresponding schematic and fluorescence images of the double-shell IMNM. All scale bars are 200 *μ*m.

Notably, by repeating the drawing procedures, IMNMs with core-shell structures could be fabricated. Specifically, the prepolymer droplet as the shell was added through the same tapered round capillary to swallow the original IMNM, as schemed in Figure S7. Wiredrawing then started, and the shell prepolymer gradually formed a conical shape, which was immediately followed by turning on the UV light. In this case, wiredrawing and solidification of the shell prepolymer happened simultaneously until the hydrogel filament finally fractured. By separately staining the core and the shell with differently colored fluorescent dyes, the core-shell structure of the resultant IMNM was clearly revealed, as shown in Figures 4(a) and 4(b). It should be mentioned that the diameter ratios of such IMNMs could be flexibly adjusted through changing the amounts of the shell prepolymer, which were directly related to the flow rates of the capillary microfluidic device (Figure S8). Besides, IMNMs with more than one shell could be generated simply via repetition of the drawing (Figure 4(c)). In addition, we chose glass, wood, polydimethylsiloxane (PDMS), and polyethylene (PE) as the substrates and successfully built needle layers on them, indicating that the microfluidic drawing method was widely applied to diverse types of substrates and that this strategy was highly versatile (Figure S9).

**Figure 5:**
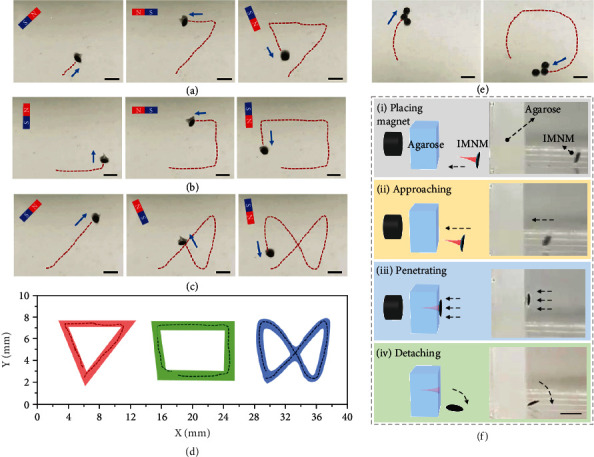
Magnetic control, penetration, and separation tests. (a–c) The IMNM following triangle, square, and bow-tie-shape trajectories under the guidance of a magnet. (d) Plotted trajectories of IMNMs on a coordinate axis. (e) Multiple IMNMs simultaneously following a circular trajectory guided by a magnet. (f) Schematics and digital images of the IMNM being attracted by the magnet (i), approaching the agarose block (ii), penetrating into the agarose (iii), and detaching from the agarose after ~15 min (iv). All scale bars are 2 mm.

Ascribed to the superior magnetic properties of the NdFeB particles encapsulated in the basement layers, IMNMs were endowed with polarities after being magnetized and their motions could be guided under magnetic control. Notably, such magnetic polarity made the needle tips of IMNMs always point to the magnetic field direction. To demonstrate the magnetic responsiveness, the IMNMs were directed to move along planned orbits by a strong magnet. It was found that the IMNM could follow differently shaped paths, including the triangle, square, and bow-tie shape, by shifting the position of the magnet or rotating the magnet to change the magnetic field direction (Figures 5(a)–5(c)). Through plotting these trajectories on a coordinate axis, the direction, route, and range of the locomotion were obviously displayed, as shown in Figure 5(d). In addition to controlling a single IMNM, a magnet could manipulate multiple IMNMs to move along a circular path at the same time, as seen in Figure 5(e).

Since the IMNMs would have direct contacts with intestinal tissues, their biocompatibility and biosafety should be guaranteed. For this purpose, 24-well plates and transwells were employed. In this coculture system, fibroblasts (NIH 3T3 cells) were seeded on the plate wells, while IMNMs were placed on the membranes of the transwell chambers, as schemed in Figure S10a. To quantify the cell viability, CCK-8 assays of the experimental group (cocultured with IMNMs) and the control group (without IMNMs) were conducted on day 1, day 2, and day 3, respectively. Results revealed that cell growth in the experimental group had negligible difference with that in the control group, demonstrating the cytocompatibility of such IMNMs (Figure S10b). Besides, hemolysis tests of these IMNMs were also executed, which had an optimistic finding of the average hemolysis rate below 1.5% (Figure S11). Additionally, GelMA, the major component of the needle layer that would be left inside intestinal tissues, has been proven by previous studies to gradually degrade for weeks or months after implantation [37]. Its degradation products are safe without causing organ toxicity or inflammatory reactions. All these outcomes indicated the ideal safety of the IMNMs for *in vivo* applications.

Benefitting from the detachable middle layer constituted by dried PVA that would quickly dissolve in aqueous liquids, the basement layer could separate from the tissue-penetrated needle layer for excretion. For evaluating the middle layer degradation time as well as the basement-needle separation time, the middle layers were immersed in artificial intestinal fluids, and changes of their dry weight were recorded. The total degradation time was found to be around 40 min (Figure S12a). Besides, using agarose blocks to mimic tissues, the whole process of IMNM penetration and separation was captured, as shown in Figure 5(f). To describe in detail, attracted by a magnet, the IMNM quickly approached the agarose and achieved penetration. Then, as the middle layer dissolved, the attachment between the basement and the needle layer weakened, and thus, the basement fell off with the needle layer remaining in the agarose after magnet removal in about 15 min. To prove the penetration ability of IMNMs, vertical and cross-section views of an agarose block with a needle layer inside were taken, as exhibited in Figure S13. It should be mentioned that dry weights of basement layers kept almost unchanged when they were immersed in artificial intestinal fluids for 48 h, indicating that there was negligible NdFeB particle leakage during the intestinal circulation and that the basements could be safely discharged (Figure S12b).

**Figure 6:**
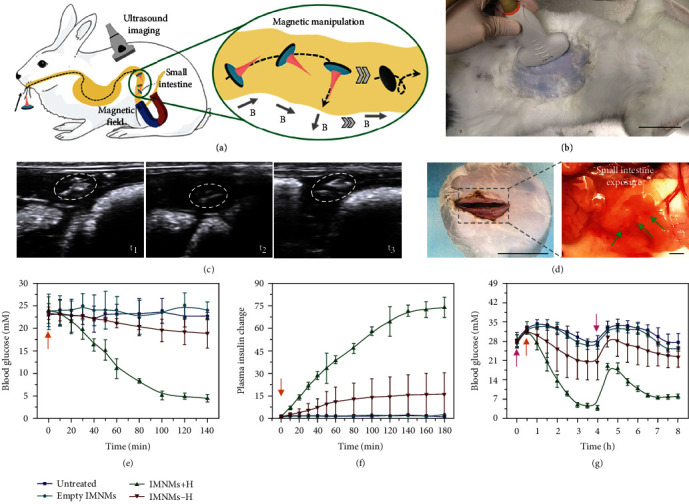
In vivo animal experiments. (a) Schematic illustration of an IMNM being manipulated by magnetic fields inside the rabbit small intestine. (b) Image of ultrasound imaging on the rabbit. Scale bar: 6 cm. (c) Ultrasound images showing the position changes of IMNMs under changing magnetic fields. White circles indicate the IMNMs. (d) Images of the exposed rabbit small intestine showing the reserved needle layers. The needles are dyed red and pointed out by green arrows. Scale bars: 6 cm (left) and 2 mm (right). (e) Blood glucose levels at different time points of diabetic rabbits receiving no treatments, empty IMNMs without insulin, insulin-loaded IMNMs under the guidance of magnetic fields, and insulin-loaded IMNMs without magnetic fields. All treatments begin at 0 min. (f) Relative plasma insulin concentrations of diabetic rabbits in the above four groups. The initial plasma insulin concentration of each group is set as the unit. All treatments begin at 0 min. (g) Longer-time blood glucose level monitoring of diabetic rabbits in the above four groups. The pink arrows indicate glucose intake, and the orange arrows indicate IMNM application.

The rapid solubility of middle layers was further tested by placing round PVA films (diameter: 1 cm) under the skin of anesthetized rats and recording the dry weight changes (Figure S14). The PVA films were found to lose intact shapes and become almost invisible after 30 min implantation, indicating the quick dissolving process in physiological conditions. After these assessments, the *in vivo* magnetic manipulation, penetration, and detachment of these IMNMs were then investigated. For this purpose, the IMNMs were placed into the rabbit small intestine via an overtube through the stomach. Then, an external magnetic field was applied to remotely steer the IMNMs, and their real-time positions were monitored by ultrasound imaging, as schematically shown in Figures 6(a) and 6(b). It was observed that the IMNMs changed their positions and moved along the small intestine under the guidance of the magnetic field (Figure 6(c)). Notably, after the operation, the small intestine was exposed, and tissue samples were collected, where traces of the reserved needle layers were found, indicating the successful penetration and detachment (Figure 6(d)). In addition, confocal laser scanning microscopy images of the needle-penetrated tissue showed that the penetration depth was more than 240 *μ*m (Figure S15). These satisfactory *in vivo* behaviors laid the foundation for the intestinal drug delivery applications of the IMNMs.

To find out the intestinal drug delivery capacity of these IMNMs, insulin was encapsulated into the needles of the IMNMs for treating diabetic rabbits. This animal model was chosen because of the high incidence of diabetes and the clinical value of oral insulin delivery. Also, the feature of delivering drugs to a general area of small intestines made the IMNMs suitable for this model. Before applying to rabbits, the drug release profile of IMNM needles was first detected *in vitro*. It was found that insulin release was concentrated in the initial 2 h at a high speed (Figure S16). After that period, the release rate slowed down and gradually approached the plateau. For the animal experiment, the diabetic rabbits were randomly formed into four separate groups, with the first group receiving no treatments (untreated), the second receiving IMNMs without insulin (empty IMNMs), the third receiving insulin-loaded IMNMs under the guidance of magnetic fields (IMNMs+H), and the fourth receiving insulin-loaded IMNMs without magnetic fields (IMNMs-H). After these treatments, the blood glucose levels of rabbits in the IMNM+H group declined to normal, and their plasma insulin levels accordingly went up, indicating the ideal therapeutic effects of these IMNMs (Figures 6(e) and 6(f)). On the contrary, both the blood glucose and plasma insulin of rabbits in the untreated group and the empty IMNM group remained almost unchanged. For the IMNM-H group, it was difficult for IMNMs to penetrate into the intestinal tissue without magnetic fields, which led to insignificant blood glucose decrease. Additionally, the magnetically guided insulin-loaded IMNMs could effectively reduce the elevated blood glucose caused by glucose ingestion and help sustain the normoglycemic state for a relatively long time. This was proven by the lower increase in the blood glucose level after glucose intake and the quicker recovery to normal of rabbits in the IMNM+H group compared to the other groups (Figure 6(g)). These results demonstrated the potential of the IMNMs in sustainable blood glucose control and treatment effect maintenance.

## 3. Discussion

In summary, using a microfluidic-based dip-printing strategy, we designed and fabricated an IMNM with a drug-loaded single-needle layer, a quickly separable middle layer, and a magnetically controllable basement for effective oral drug administration. The oblate basements and middle layers were first generated layer by layer through directly writing pregel droplets on a shifting slide. Owing to the simple, controllable capillary devices and microfluidics, these basements and middle layers displayed satisfactory uniformity and could be mass-produced. By then adding a viscous droplet on the middle layer, and wiredrawing it while solidifying it, the conical needle layer could be easily fabricated. Notably, via this approach, the needle layer could be constructed on substrates with various properties, including hydrogels, wood, plastics, glass, etc., and IMNMs with multiple shells could be fabricated, revealing the versatility of this approach. During oral administration, IMNMs could be encapsulated in commercial enteric capsules. These capsules stayed intact and protected the inner IMNMs through the stomach; while they disintegrated and released IMNMs in the small intestine. Once released, the basements of IMNMs enabled the magnetically guided movements due to magnetic polarity, and the needle layers realized the intestinal mucosa penetration and drug delivery based on appropriate lengths and hardness. The middle layers, which were composed of dissolvable PVA, would rapidly dissolve, helping the separation between basements and needle layers, promoting sustainable drug release from the needles, and leading to excretion of the basements. The practical values of such IMNMs were further demonstrated by using them to orally deliver insulin to regulate blood glucose levels of diabetic rabbit models.

Future development of this dip-printing method and these IMNMs can be the enrichment of materials, the diversification of structures, and the expansion of applications. With respect to the materials, in addition to the presented UV-crosslinking GelMA/PVA hydrogels, other viscous materials such as heat-solidifying polymers (e.g., PDMS) or drying-curing biomaterials (e.g., hyaluronic acids) can also be the candidates for the needle layer. Besides, stimulus-sensitive hydrogels such as sodium alginate and functional molecules such as cell-penetrating peptides can be introduced, which can help IMNMs overcome the physiological barriers of gastrointestinal tracts. Additionally, by further adjusting the parameters and operation details of the wiredrawing process, the needle layer may be imparted with diverse structures, such as the rugby shape, the filament shape, and the rosary bead shape. These architectures may enhance the intestinal adhesion of IMNMs and facilitate pharmaceutical absorption. Moreover, these micromotors can carry a variety of substances for many kinds of applications. For example, by carrying different drugs, they can achieve oral drug therapies for different diseases. Also, by being loaded with luminous particles, they can provide a feasible approach for detection and exploration of the surrounding environments.

## 4. Materials and Methods

### 4.1. Viscosity Tests and Mechanical Tests

The viscosity tests of the prepolymer solutions were conducted via a rotational rheometer (Anton Paar MCR302). To evaluate the influence of PVA concentrations, gradient solutions of 5 wt% GelMA mixed with 0 wt%, 5 wt%, 10 wt%, 15 wt%, 20 wt%, or 25 wt% PVA were prepared. To evaluate the influence of GelMA concentrations, gradient solutions of 15 wt% PVA mixed with 0 wt%, 10 wt%, 20 wt%, 30 wt%, or 40 wt% GelMA were prepared. The number of parallel trials for each group was three. The mechanical tests of the solidified DN hydrogels were carried out using an electronic material testing system (INSTRON). For the tests, gradient hydrogels with PVA concentrations ranging from 0 wt% to 25 wt% or GelMA concentrations ranging from 0 wt% to 40 wt% were generated. The number of parallel trials for each group was three.

### 4.2. Fabrication and Characterization of the Double-Layer Composite

A mixed solution of 80 vol% PEGDA, 1 vol% HMPP, and 30 wt% NdFeB was first pumped from a 1 mL syringe into a tapered round capillary with the flow rate of 6 *μ*L/min. A clean glass plate was placed below the capillary to collect the pumped solution, and its position was changed every 3 s to generate an oblate droplet array. After UV irradiation (365 nm) for 30 s, the oblate droplets were solidified and the magnetic basements could be obtained. Notably, to facilitate the dispersion of NdFeB particles, the mixed solution was oscillated violently both before and during the printing process. Then, a solution of 30 wt% PVA (M_w_ 13000-23000) was pumped through another tapered round capillary with the same flow rate and added on the basements to form covers. The PVA covers were dried at room temperature overnight to constitute the middle layers. For characterization, the optical images were taken by a CCD camera (Oplenic Digital Camera, Hangzhou, China) attached to a stereomicroscope (JSZ6S, Jiangnan Novel Optics, Nanjing, China). The digital images were captured via a Huawei P30 pro mobile phone. The SEM images were taken using a field emission scanning electron microscope (FESEM, Ultra Plus, Zeiss) and recolored by Photoshop CC 2018.

### 4.3. Fabrication and Characterization of IMNMs

A viscous prepolymer solution composed of 30 wt% GelMA, 15 wt% PVA (M_w_ 89000-98000), and 1 vol% HMPP was first prepared. A droplet of the prepolymer solution was extruded through a tapered round capillary of a microfluidic device. Then, the below glass plate carrying the double-layer composites gradually approached the capillary until the composite and the droplet contacted. After that, the glass plate moved away from the capillary to wiredraw the droplet and make it conical. At this moment, the UV light was turned on to realize the synchronous drawing and solidifying. The process continued until the polymer filament broke up to generate the needle layer. Finally, the resultant IMNM was frozen (-25°C, 20 min) and thawed (room temperature, 20 min) for three times. To fabricate IMNMs with core-shell structures, another prepolymer droplet as the shell was added through the same capillary and engulfed the original IMNM. The drawing then began, and the UV light was on as soon as the shell droplet formed a conical shape. Through simultaneous wiredrawing and solidifying, the shell filament fractured and the core-shell IMNM was generated, which also experienced the freeze thawing process. By repeating such drawing procedures, IMNMs with multiple shells could also be created. The fluorescence images of the IMNMs were recorded by a fluorescent confocal microscope (Olympus, FV3000).

### 4.4. In Vitro Manipulation of IMNMs via a Magnet

Before the magnetic manipulation, the IMNMs were magnetized by an impulse magnetizer (J302, Jinlang Co. Ltd., Nanjing, China). The output voltage was set as 600 V, and the magnetization times were three. To guide the IMNMs along specific trajectories, a cylinder magnet (3 cm in diameter and 3 cm in height) was placed under the IMNMs and moved along these determined patterns. According to previous studies [38–40], a 5 wt% agarose block was placed in a water container together with a IMNM for mimicking the penetration and separation of IMNMs *in vitro*. The cylinder magnet was put on the other side of the agarose block to attract the IMNM and induce penetration. After less than 20 min, the magnet was withdrawn, and the needle layer and the magnetic basement separated.

### 4.5. Artificial Intestinal Fluid Immersion Tests

The middle layers were prepared by drying 100 *μ*L of 30 wt% PVA at room temperature. Their original weights were first measured, and they were then immersed in artificial intestinal fluids at 37°C. The dry weights of these middle layers were recorded every 6 min. In addition, the magnetic basements were fabricated by solidifying 100 *μ*L of the mixture of 80 vol% PEGDA, 1 vol% HMPP, and 30 wt% NdFeB via UV exposure. These magnetic basements were then immersed in artificial intestinal fluids at 37°C, and their dry weights were detected from 0 h to 48 h. The number of parallel trials for each group was three.

### 4.6. In Vivo Manipulation of IMNMs

The male New Zealand rabbits (2.5-3 kg) were provided by Kaisijia Biotech Co., Ltd. (Nanjing, China). All animals were treated in strict accordance with the Beijing Administration Rule of Animals in China and have received approval from the Animal Investigation Ethics Committee of Drum Tower Hospital. Before the operation, the animals were fasted for 24 h. Anesthesia was performed by intraperitoneal injection of 3% sodium pentobarbital at the dose of 45 mg/kg body weight. IMNMs (500 *μ*m in height) were put directly to the rabbit small intestine via an overtube and were steered by an external magnetic field pointing outwards. The position changes of the IMNMs were recorded by ultrasound imaging (Mindray, M550). After the rabbit was sacrificed, the IMNM-penetrated intestinal tissue was collected and observed under a confocal laser scanning microscope (Olympus, FV3000).

### 4.7. Modelling and Treatment of Diabetes in Rabbits

Before modelling, all rabbits experienced fasting treatment for 8 h. To establish the diabetic rabbit model, 5 wt% alloxan was injected through the rabbit auricular vein at the dose of 150 mg/kg body weight in less than 30 s. Food was provided immediately after the injection. After 3 d, the blood glucose levels of the rabbits were found to be above 16 mM. The rabbits were then stochastically divided into four groups: the first received no treatment (untreated), the second received IMNMs without insulin (empty IMNMs), the third received insulin-loaded IMNMs under the guidance of magnetic fields (IMNMs+H, insulin content: 6 U/kg body weight), and the fourth received insulin-loaded IMNMs without magnetic fields (IMNMs-H). At designed time points, the blood glucose and the plasma insulin were monitored from the auricular vein via a glucometer (Yuwell 305A, Shanghai, China) and a Human Insulin ELISA kit (Beyotime Biotechnology, Shanghai, China), respectively. The number of parallel trials for each group was three. Besides, the glucose was consumed by injecting 5% glucose into the auricular vein.

## Data Availability

All data are available in the main text or the supplementary materials.

## Supplementary Materials

Supplementary Materials Materials and methods Figure S1: size control of the magnetic basements. Figure S2: EDS results of the cross-section of the basement layer. Figure S3: gelation analysis. Figure S4: factors that influence the viscosity of prepolymer solutions of GelMA-PVA mixture. Figure S5: factors that influence the mechanical strength of the solidified DN hydrogels. Figure S6: force changes with displacements to show the maximum stress that an IMNM can tolerate. Figure S7: schematic illustrations of fabricating core-shell IMNMs. Figure S8: shell thickness control of core-shell IMNMs. Figure S9: independent microneedle built on different basements. Figure S10: cytocompatibility of IMNMs. Figure S11: digital image and quantifiable data of hemolysis tests of IMNMs. Figure S12: artificial intestinal fluid immersion tests. Figure S13: digital images of IMNMs penetrating in the agarose. Figure S14: dissolving processes of PVA films under rat skins. Figure S15: confocal laser scanning microscopy images of the IMNM needle left inside the rabbit small intestine tissue. Figure S16: in vitro release of FITC-insulin from needle layer materials.
