# Supplementary material for: Dip-Printed Microneedle Motors for Oral Macromolecule Delivery
Source: Research (Wash D C). 2022 Jul 20;2022:9797482. doi: 10.34133/2022/9797482 (PMC9343079; doi:10.34133/2022/9797482)
Supplement: Supplementary Materials — Materials and methods Figure S1: size control of the magnetic basements. Figure S2: EDS results of the cross-section of the basement layer. Figure S3: gelation analysis. Figure S4: factors that influence the viscosity of prepolymer solutions of GelMA-PVA mixture. Figure S5: factors that influence the mechanical strength of the solidified DN hydrogels. Figure S6: force changes with displacements to show the maximum stress that an IMNM can tolerate. Figure S7: schematic illustrations of fabricating core-shell IMNMs. Figure S8: shell thickness control of core-shell IMNMs. Figure S9: independent microneedle built on different basements. Figure S10: cytocompatibility of IMNMs. Figure S11: digital image and quantifiable data of hemolysis tests of IMNMs. Figure S12: artificial intestinal fluid immersion tests. Figure S13: digital images of IMNMs penetrating in the agarose. Figure S14: dissolving processes of PVA films under rat skins. Figure S15: confocal laser scanning microscopy images of the IMNM needle left inside the rabbit small intestine tissue. Figure S16: in vitro release of FITC-insulin from needle layer materials. [file 9797482.f1.docx]

Supplementary Materials for

**Dip-Printed Microneedle Motors for Oral Macromolecule Delivery**

Xiaoxuan Zhang, Guopu Chen, Lijun Cai, Lu Fan, Yuanjin Zhao*

*Corresponding author. Email: yjzhao@seu.edu.cn

**Supplementary materials and methods**

*Materials*: Polyethylene glycol diacrylate (PEGDA, average M_n_ 700), polyvinyl alcohol (PVA, M_w_ 89000-98000 and M_w_ 13000-23000), and 2-hydroxy-2-methylpropiophenone (HMPP) were bought from Sigma-Aldrich. Methacrylate Gelatin (GelMA, MA 97%) and agarose were purchased from Aladdin. Neodymium-Iron-Boron (NdFeB) microparticles (5 µm) were obtained from XND Co. Ltd. (Guangzhou, China). TetraSpeck™ microspheres (0.1 µm, fluorescent blue/green/orange/dark red) were from Invitrogen. Alloxan was provided by FeiYuBio Co. Ltd. (Nantong, China). Fluorescein isothiocyanate labelled insulin (FITC-insulin) and human recombinant insulin were offered by RuiXiBio Co. Ltd. and the Drum Tower Hospital, respectively. High Glucose Dulbecco’s Modified Eagle’s Medium, fetal bovine serum, and penicillin-streptomycin double antibiotics were bought from Gibco. Artificial intestinal fluids (pH = 6.8) and PBS buffer solutions (pH = 7.4) were self-prepared at the laboratory.

*Experimental setups for CCK-8 assays*: NIH 3T3 cells were provided by the Cell Bank of the Chinese Academy of Sciences (Shanghai, China). The components of the complete culture medium were 89 vol% High Glucose Dulbecco’s Modified Eagle’s Medium, 10 vol% fetal bovine serum, and 1 vol% penicillin-streptomycin double antibiotics. To conduct the CCK-8 assays, 24-well plates and transwells were used. NIH 3T3 cells were seeded on the plate wells. For the experimental group, IMNMs were placed on the membranes of the transwell chambers; while for the control group, no additional operations were performed. For three consecutive days, the initial culture media were withdrawn and new culture media mixed with 10 vol% CCK-8 solution were added. 3 h later, 100 µL of the culture media were transferred to a 96-well plate. There were six parallels for each group on each day. To compare the cell viability, the OD values of the culture media at 450 nm were detected by a multimode plate reader (SYNERGY|HTX).

*Hemolysis tests*: Male Sprague Dawley (SD) rats (250 g) were provided by Kaisijia Biotech Co., Ltd (Nanjing, China). Animals were treated in strict accordance with the Beijing Administration Rule of Animals in China and have received approval from Animal Investigation Ethics Committee of the Drum Tower Hospital. Fresh whole blood was collected from the rat tail in the EDTA anticoagulant tube. The 2% blood cell solution was acquired by repeatedly washing the whole blood sample and re-dispersing the precipitation in PBS buffer solution. To prepare the material leaching liquor, the IMNMs were immersed in PBS buffer solution overnight in advance. Then the material leaching liquor and the blood cell solution were mixed at the proportion of 1:1. Using the same mixing method, deionized water and PBS buffer solution were set as the positive and the negative control, respectively. The number of parallel trials for each group was six. All the groups were incubated at 37℃ for 4 h. After centrifugation at 3000 rpm for 5 min, the OD values of the supernatants at 570 nm were measured by the multimode plate reader. The hemolysis rate could be calculated by the following formula:

$$\text{Hemolysis}\left( \text{\%} \right)\text{=}{\text{(}\text{OD}_{\text{sample}}\text{-}\text{OD}_{\text{negative}}\text{)}}/{\text{(}\text{OD}_{\text{positive}}\text{-}\text{OD}_{\text{negative}}\text{)}}$$

*In-vitro insulin release tests*: FITC-insulin was added to the needle layer prepolymer with the final concentration of 0.5 mg/mL. These prepolymers were solidified into same-sized polymer blocks by UV irradiation. They were then immersed in PBS buffer solutions and incubated in a thermomixer (37℃, 300 rpm). The number of parallel trials was six. At specific time points, the fluorescence intensity of the PBS buffer solution was detected using the multimode plate reader (excitation wavelength: 495 nm, emission wavelength: 550 nm). Based on the standard curve of FITC-insulin, the fluorescence intensity data could be converted to concentration information. The total release amount could be calculated using the below formula:

$$\text{Cumulative release}\left( \text{\%} \right)\text{=}\frac{\text{Concentration}_{\text{FITC insulin}}\text{×}\text{Volume}_{\text{PBS solution}}}{\text{0.5 mg/mL×}\text{Volume}_{\text{polymer blocks}}}\text{×100\%}$$

**Supplementary Figures**

**
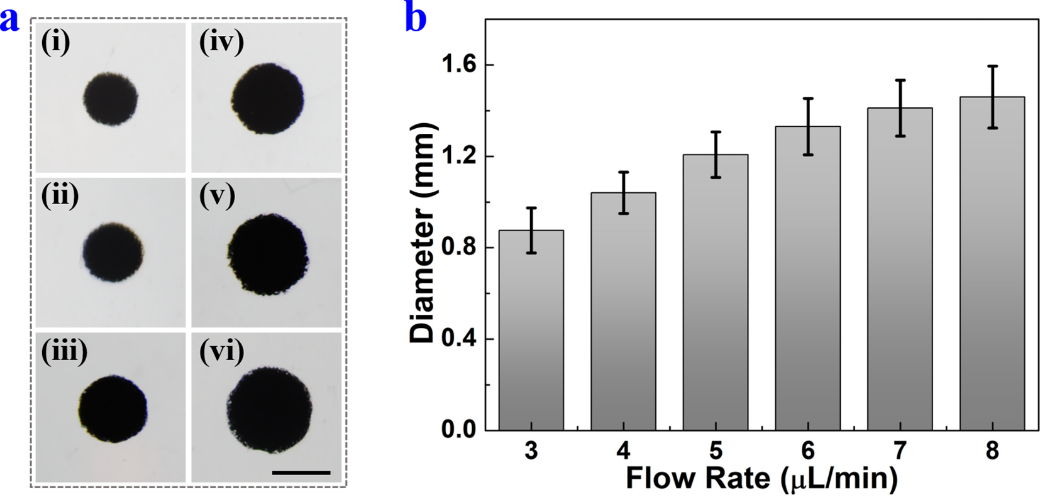
**

**Fig. S1. Size control of the magnetic basements.** (**a**) Optical images of magnetic basements with different sizes. Scale bar: 1 mm. (**b**) Statistic relationship between the diameter of magnetic basements and the flow rate of the capillary microfluidic device.

**
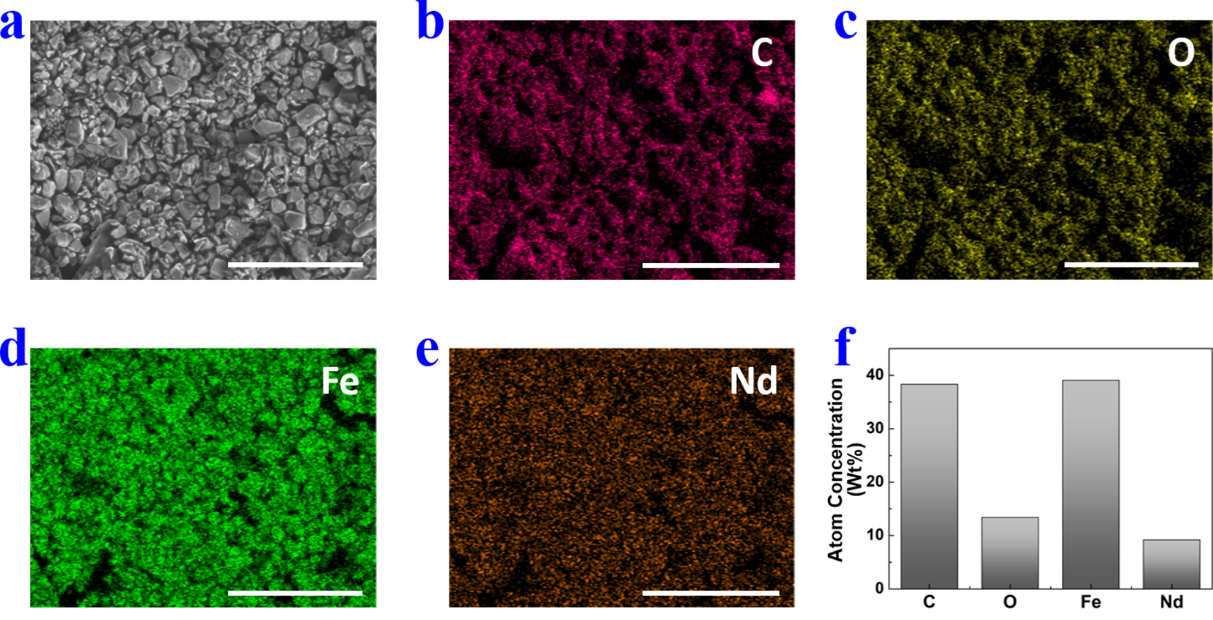
**

**Fig. S2. EDS results of the cross section of the basement layer.** (**a**) SEM image. (**b-e**) EDS elemental mapping. (**f**) Corresponding atom concentrations. Scale bars: 50 µm.

**
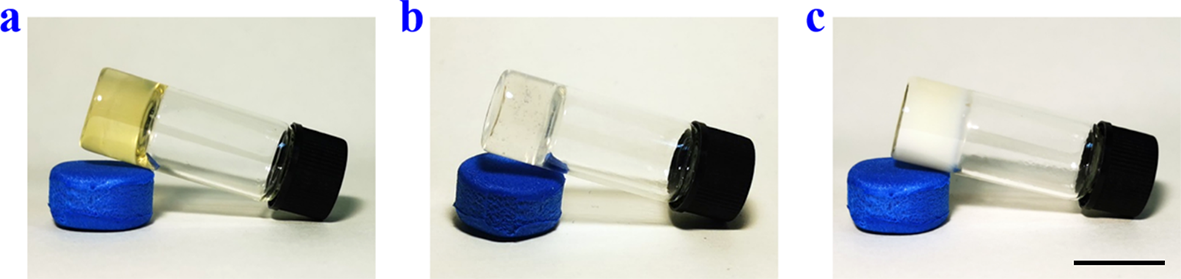
**

**Fig. S3. Gelation analysis.** (**a**) Digital image of 20 wt% GelMA after UV irradiation. (**b**) Digital image of 10 wt% PVA after freeze thawing. (**c**) Digital image of the DN hydrogels that consist of 20 wt% GelMA and 10 wt% PVA. Scale bar: 1 cm.

**
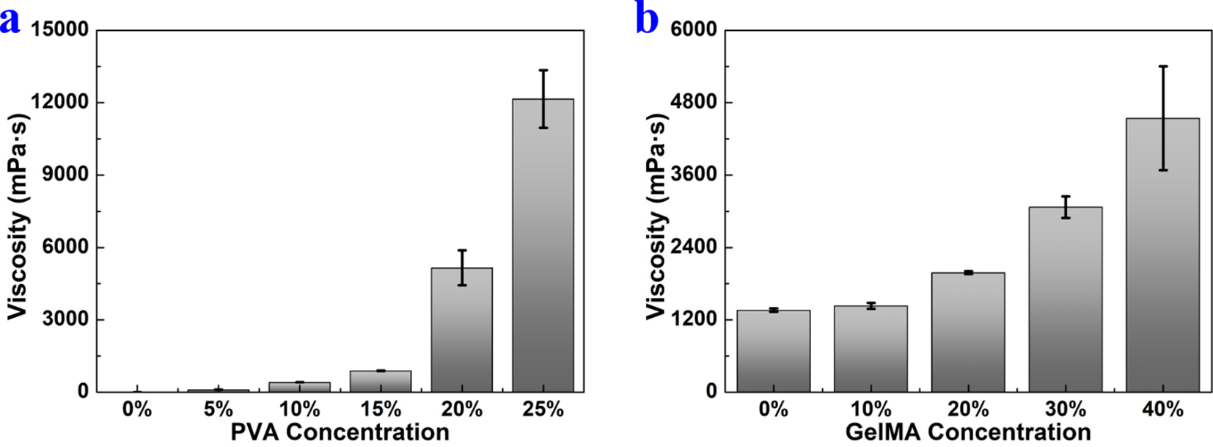
**

**Fig. S4. Factors that influence the viscosity of prepolymer solutions of GelMA-PVA mixture.** (**a**) Statistical relationship between the viscosity and the PVA concentration. (**b**) Statistical relationship between the viscosity and the GelMA concentration.

**
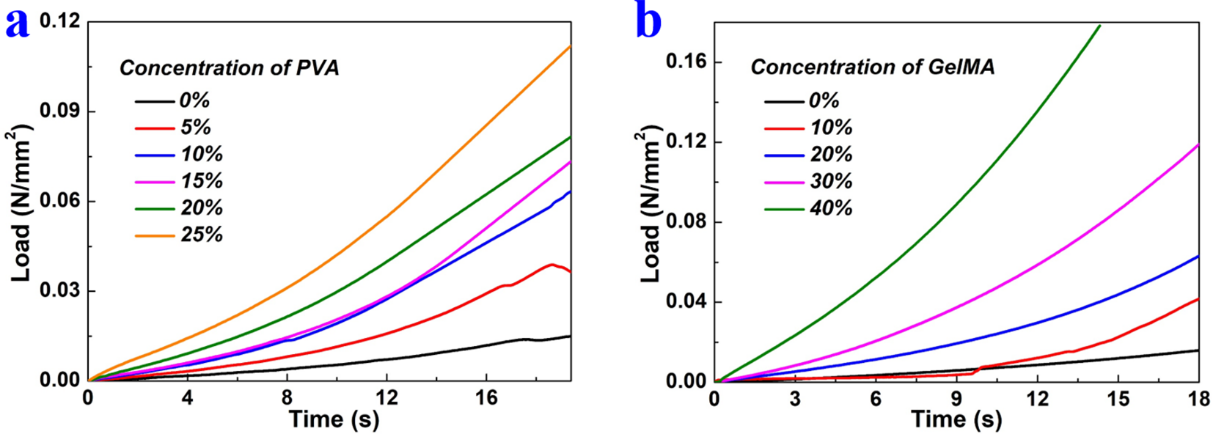
**

**Fig. S5. Factors that influence the mechanical strength of the solidified DN hydrogels.** (**a**) Stress curves of the DN hydrogels at different PVA concentrations. (**b**) Stress curves of the DN hydrogels at different GelMA concentrations.


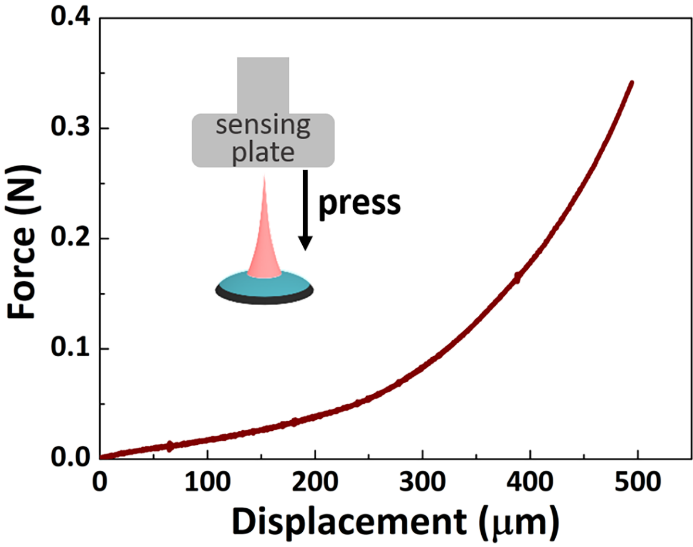


**Fig. S6.** Force changes with displacements to show the maximum stress that an IMNM can tolerate.

**
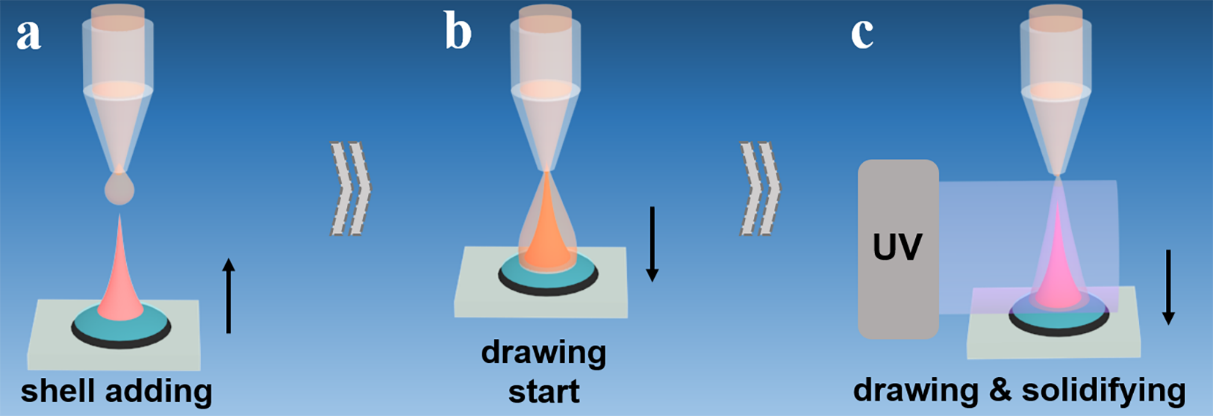
**

**Fig. S7.** **Schematic illustrations of fabricating core-shell IMNMs.** (**a**) A droplet is added to engulf the original IMNM and serve as the shell. (**b**) Wiredrawing starts and the shell forms a taper shape. (**c**) UV light is on, making wiredrawing and solidifying happen simultaneously until breaking up.

**
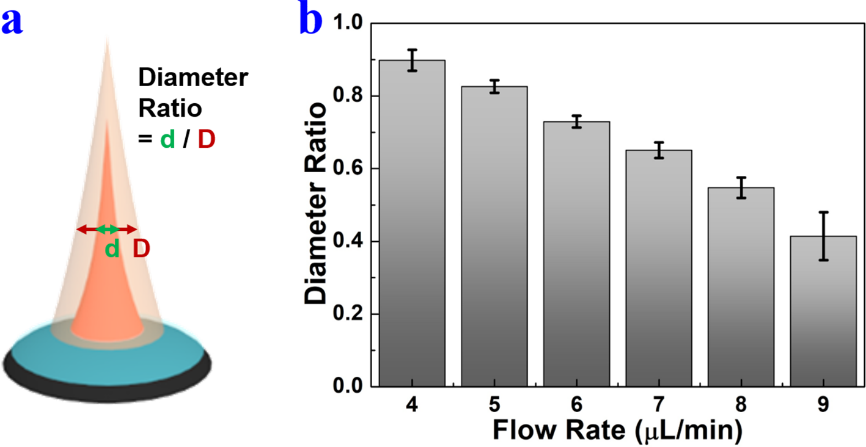
**

**Fig. S8. Shell thickness control of core-shell IMNMs.** (**a**) Schematic illustration pointing out the diameter ratio. (**b**) Statistic relationship between the diameter ratio and the flow rate of the capillary microfluidic device.

**
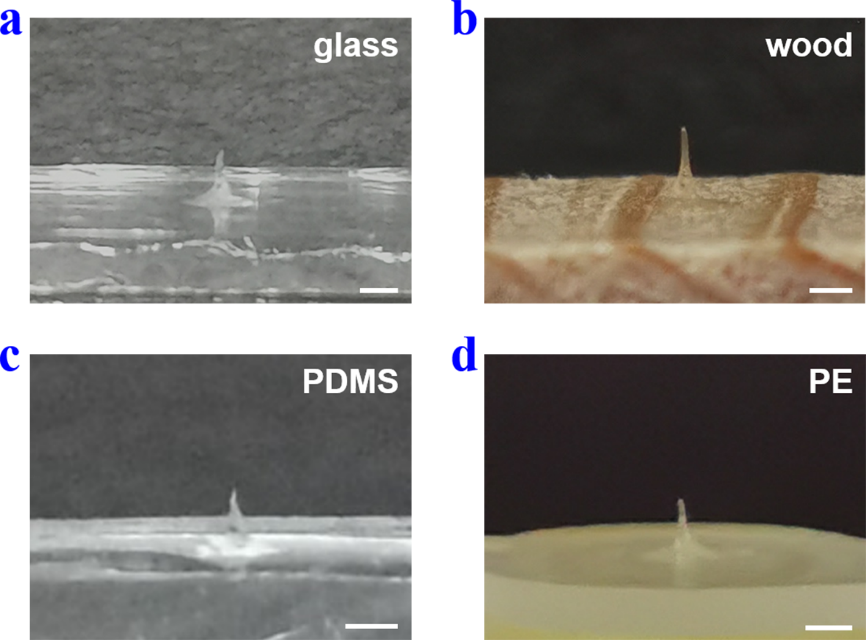
**

**Fig. S9. Independent microneedle built on different basements.** The basements are made of glass (**a**), wood (**b**), PDMS (**c**), and PE (**d**). All scale bars: 500 μm.

**
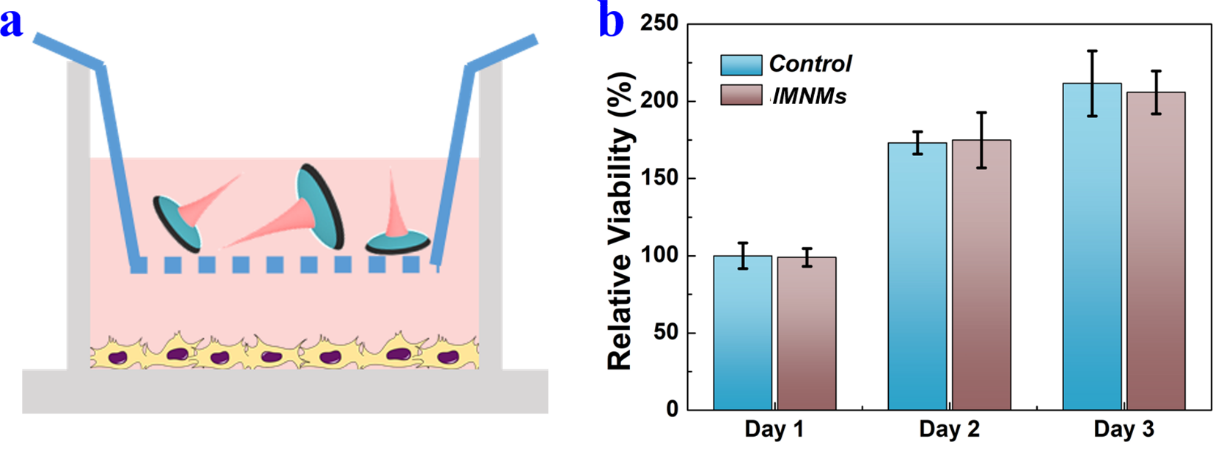
**

**Fig. S10. Cyto-compatibility of IMNMs.** (**a**) Schematic illustration of NIH 3T3 cells and IMNMs co-cultured in transwells. (**b**) CCK-8 results showing the viability of cells cultured without IMNMs (control) or with IMNMs on day 1, day 2, and day 3.

**
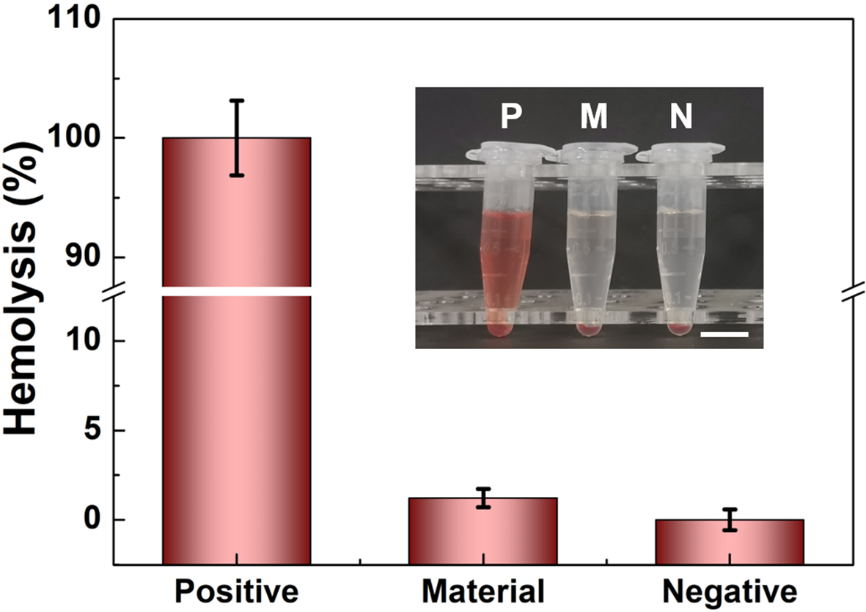
**

**Fig. S11. Digital image and quantifiable data of hemolysis tests of IMNMs.** Positive control (P) is deionized water. Negative control (N) is fresh PBS solution. Scale bar: 1 cm.

**
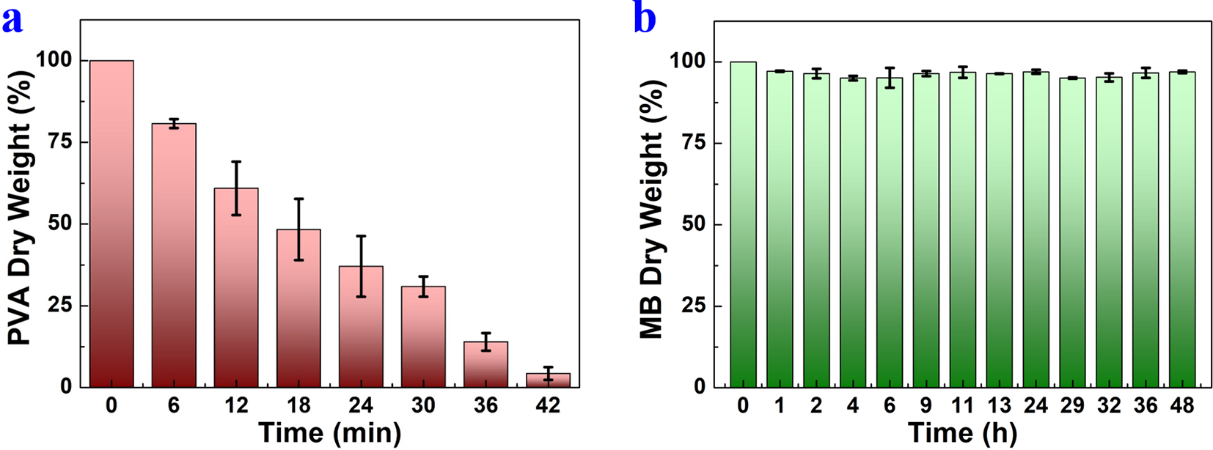
**

**Fig. S12. Artificial intestinal fluid immersion tests.** (**a**) Dry weight changes of PVA middle layers showing their degradability. (**b**) Dry weight changes of magnetic basement (MB) layers showing their stability.

**
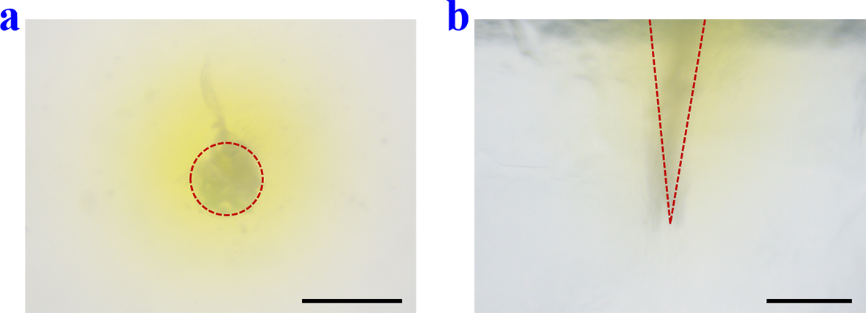
**

**Fig. S13. Digital images of IMNMs penetrating in the agarose.** (**a**) The vertical view. (**b**) Cross section view. The needle layer of the IMNM is dyed yellow. Both scale bars: 300 μm.

**
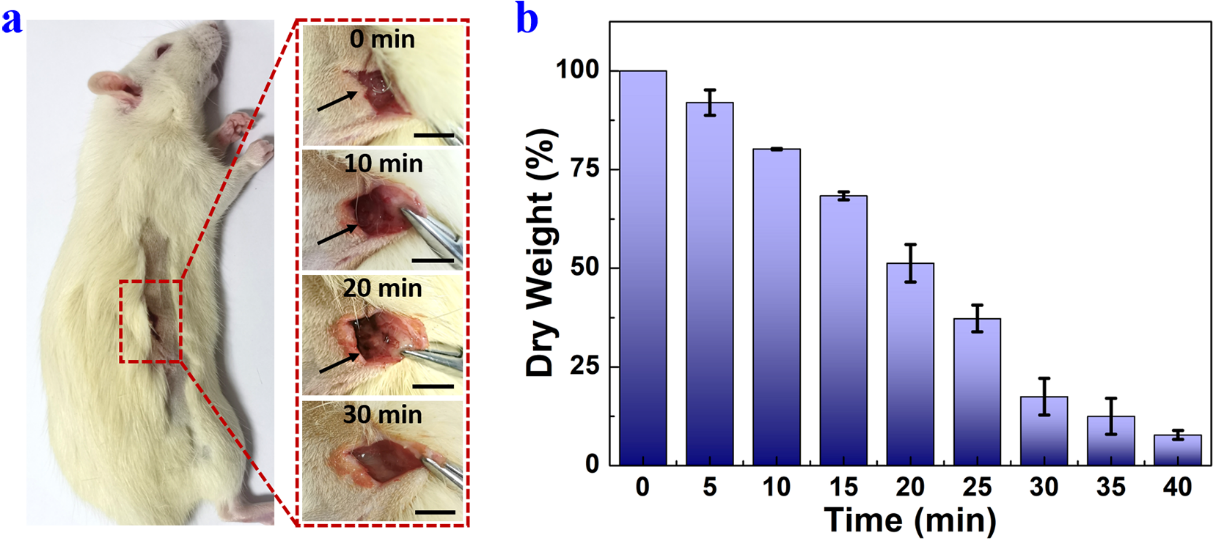
**

**Fig. S14. Dissolving processes of PVA films under rat skins.** (**a**) Digital images of the PVA film over time. The black arrows point to the PVA film. Scale bar: 1 cm. (**b**) Dry weight changes of PVA films.

**
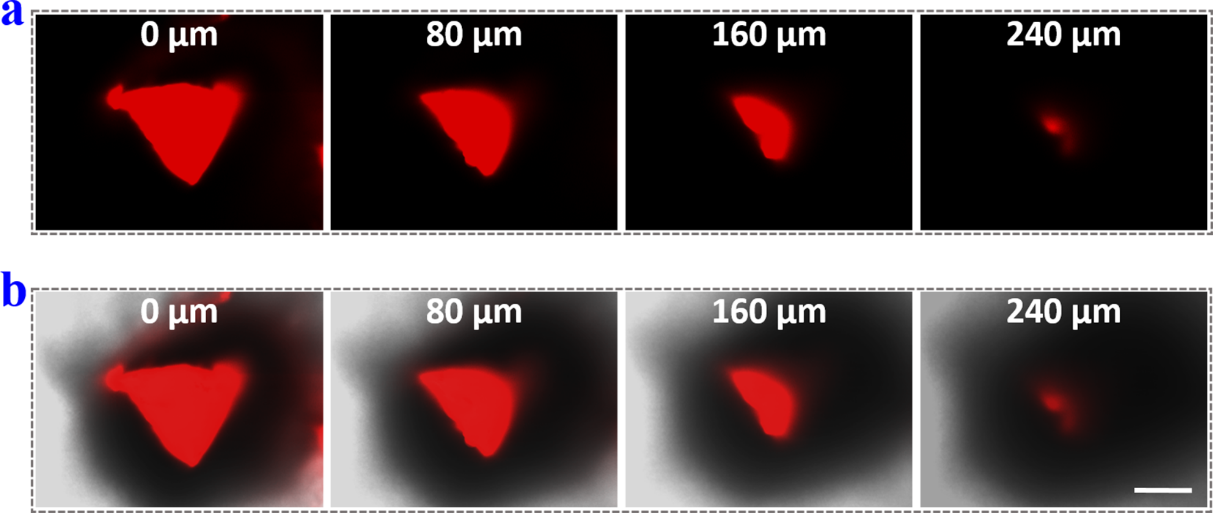
**

**Fig. S15. Confocal laser scanning microscopy images of the IMNM needle left inside the rabbit small intestine tissue.** (**a**) The fluorescence field. (**b**) The superposition of fluorescence field and bright field. The images vary in depths: 0 μm, 80 μm, 160 μm, 240 μm. Scale bar: 100 μm.

**
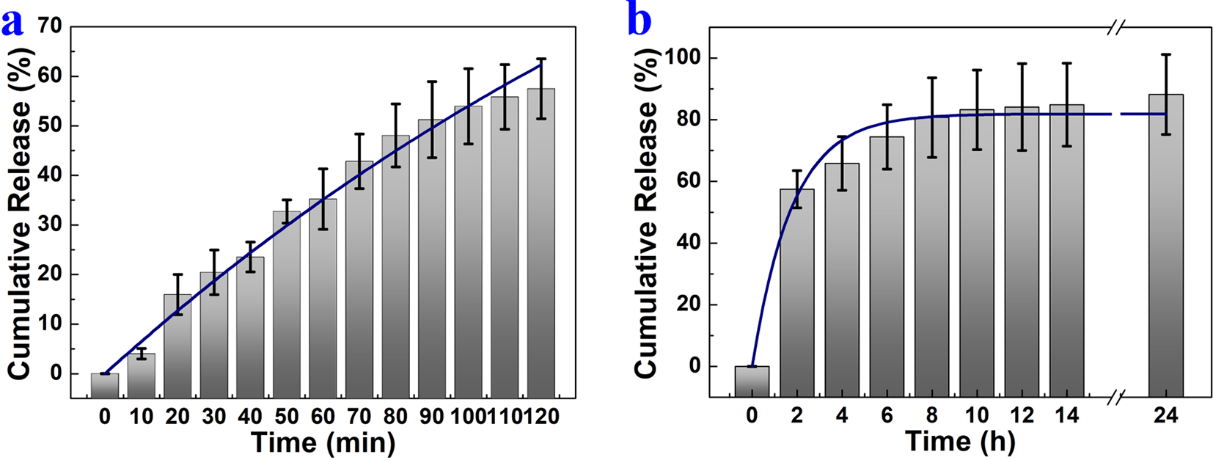
**

**Fig. S16. *In-vitro* release of FITC-insulin from needle layer materials.** (**a**) The release profile in 120 min. (**b**) The release profile in 24 h.
